# Red Algal Phylogenomics Provides a Robust Framework for Inferring Evolution of Key Metabolic Pathways

**DOI:** 10.1371/currents.tol.7b037376e6d84a1be34af756a4d90846

**Published:** 2016-12-02

**Authors:** Huan Qiu, Hwan Su Yoon, Debashish Bhattacharya

**Affiliations:** Department of Biological Sciences, Sungkyunkwan University, Suwon, South Korea

## Abstract

Red algae comprise an anciently diverged, species-rich phylum with morphologies that span unicells to large seaweeds. Here, leveraging a rich red algal genome and transcriptome dataset, we used 298 single-copy orthologous nuclear genes from 15 red algal species to erect a robust multi-gene phylogeny of Rhodophyta. This tree places red seaweeds (Bangiophyceae and Florideophyceae) at the base of the mesophilic red algae with the remaining non-seaweed mesophilic lineages forming a well-supported sister group. The early divergence of seaweeds contrasts with the evolution of multicellular land plants and brown algae that are nested among multiple, unicellular or filamentous sister lineages. Using this novel perspective on red algal evolution, we studied the evolution of the pathways for isoprenoid biosynthesis. This analysis revealed losses of the mevalonate pathway on at least three separate occasions in lineages that contain Cyanidioschyzon, Porphyridium, and Chondrus. Our results establish a framework for in-depth studies of the origin and evolution of genes and metabolic pathways in Rhodophyta.

## Introduction

Red algae (Rhodophyta) form a monophyletic lineage containing ~7,000 described species^1^ that exhibit a wide variety of morphological and ultra-structural forms and have complex reproductive strategies. The Cyanidiophytina (e.g., *Galdieria* and *Cyanidioschyzon*) include extremophiles that thrive in volcanic areas surrounding hot springs. In contrast, their mesophilic sisters (Rhodophytina) are globally distributed from freshwater environments to open oceans and deep oceans (>200 m) to the intertidal zone. Despite a highly reduced core gene inventory that resulted from an ancient phase of genome reduction^2^, red algae represent one of the few eukaryotic lineages that have evolved complex multicellularity^3^, typified by red seaweeds such as *Porphyra* and *Gracilaria*. Red seaweeds account for ~95% of known red algal taxa and are important sources of agricultural (e.g., *nori*) and industrial products (e.g., agar and carrageenan).

Studies of red algal systematics have largely relied on a handful of plastid and nuclear genes^4^^,^ 5 ^,^ 6 ^,^ 7 ^,^ 8 and focused on a broad diversity of lineages within the Florideophyceae^9^^,^ 10. One of the major findings of these analyses is the separation of Cyanidiophytina from the Rhodophytina^4^^,^ 8. Whereas Cyanidiophytina contain only two known families (Cyanidiaceae and Galdieraceae), Rhodophytina encompass six classes: Bangiophyceae, Florideophyceae, Compsopogonophyceae, Porphyridiophyceae, Rhodellophyceae, and Stylonematophyceae^4^. Excluding the well-supported monophyly of Bangiophyceae and Florideophyceae (hereafter, collectively referred to as red seaweeds), relationships among the remaining classes remain controversial^4^^,^ 5 ^,^ 6 ^,^ 7 ^,^ 8.

In this study, we applied phylogenomics to a rich genomic dataset to erect a robust red algal tree of life. The dataset encompassed 298 orthologous nuclear-encoded genes from all major red algal lineages. In contrast to previous phylogenies built using smaller datasets^4^^,^ 5 ^,^ 6 ^,^ 7 ^,^ 8, our results support a fundamental, ancient split between red seaweeds and non-seaweed lineages among mesophiles. We discuss the implication of this new perspective on red algal phylogeny to understanding the evolution of multicellularity in red algae, and demonstrate the utility of this phylogenetic framework to infer the evolution of the mevalonate (MVA) pathway of isoprenoid biosynthesis in Rhodophyta.

## Methods

**Construction of single-copy orthologous gene alignments**

We created a local database that includes protein sequences (translated from EST or predicted from genome sequences) from 15 red algal taxa^2^^,^ 11 ^,^ 12 ^,^ 13 ^,^ 14 ^,^ 15 ^,^ 16 (Fig. 1A) and 3 green algae^17^^,^ 18 ^,^ 19 (Table 1, Appendix 1). This database, after removing short sequences with length <100 amino acids, was used in a self-query using BLASTp (*e*-value cutoff = 1e-5). The BLASTp search output was used as input for OrthoMCL^20^ with parameters (evalueExponentCutoff = -10, percentMatchCutoff = 40, inflation = 1.5) to construct orthologous gene families. Among these families, we searched for single-copy orthologous genes with one gene copy per species (allowing missing data in up to three red algae and in no more than one green alga). For each orthologous gene family, the corresponding sequences were retrieved and aligned using MUSCLE (version 3.8.31) under the default settings^21^. The alignments were then trimmed using TrimAl (version 1.4)^22^ in automated mode (-automated) and then ‘polished’ with T-COFFEE (version 9.03)^23^ to removed poorly aligned residues (conservation score ≤ 5) among the aligned blocks. A total of 298 single-gene alignments (length >150 amino acids and with ≥15 sequences) were retained for downstream analysis.

**Construction of the multi-protein phylogeny**

The 298 single-copy gene alignments were concatenated into a super-protein alignment. A phylogenetic tree was inferred using Phylobayes (version 3.3)^24^ under the CAT model^25^. This is a mixture model that takes into consideration site-specific evolutionary properties (such as rate and profile) within the alignment^25^. The CAT model generally fits data significantly better than one-matrix models such as LG and WAG. We set up two chains that ran in parallel and assessed convergence periodically using ‘bpcomp’ and ‘tracecomp’ functions. Convergence assessments were done based on sampled trees (taking one from every 10 trees) following burnin equal to 20% of the entire length of the chain. The two chains were stopped when they converged to an acceptable level that allows good qualitative measurement of the posterior consensus. According to the user instructions (www.phylobayes.org/), an acceptable run corresponds to a maximum discrepancy across all bipartitions (maxdiff <0.3) when monitored with the ‘bpcomp’ function, and statistical discrepancies <0.3 and effective sizes >50 for all parameters when monitored with the ‘tracecomp’ function.

**Construction of coalescence model-based species trees**

We built a coalescence model-based red algal phylogeny with 100 replicates following Seo's method^26^. For each replicate, we randomly sampled 298 genes with replacement. For each sampled alignment, a pseudo-alignment was generated by random sampling of amino acid site from the original alignment with replacement. Only one green algal sequence (as outgroup) was retained with the priority given to *Chlamydomonas reinhardtii*, *Chlorella variabilis*, and *Micromonas *RCC299 in order. A ML tree was built for each pseudo-alignment using IQtree (version 0.9.6)^27^ under the best-fit amino acid evolutionary model selected on the fly (-m TEST). The resulting 298 ML trees, rooted with outgroup sequences, were then used for maximum pseudo-likelihood tree construction using MP-EST (version 1.4) under the default settings^28^. This procedure was repeated 100 times and the resulting 100 maximum pseudo-likelihood trees were summarized under majority rule using the ‘consense’ function in Phylip (http://evolution.genetics.washington.edu/phylip.html).

**Phylogenetic analyses of mevalonate pathway genes**

*Galdieria sulphuraria* proteins in the MVA pathway (module identifier: M00095) and the methylerythritol phosphate (MEP) pathway (module identifier: M00096) were retrieved from the KEGG database^29^ and used as queries against NCBI (nr) using BLASTp (*e*-value cutoff = 1e-5) (http://blast.ncbi.nlm.nih.gov/Blast.cgi). The representative sequences (e.g., from Metazoa and land plants) were retrieved from Genbank. Local BLASTp searches (*e*-value cutoff = 1e-5) were done against our red algal database aforementioned followed by retrieval of the significant hits. *Galdieria phlegrea* sequences were retrieved from the previous study^30^. Each *G. sulphuraria* query, together with the homologs (from Genbank and our local database), were aligned using MUSCLE (version 3.8.31)^21^ under the default settings. The alignment was trimmed using trimAl (version 1.4)^22^ in the automated mode (-automated). ML trees were built using IQtree (version 0.9.6)^27^ under the best amino acid evolutionary model selected using (-m TEST) with branch support values estimated using 1,500 ultrafast bootstrap replicates (-bb 1500). The resulting trees were manually inspected. Distantly related paralogs (if any) were removed manually and the trees were rebuilt following the procedure described above.

**Validation of gene losses in red algae****

We searched for the *G. sulphuraria* MVA and MEP proteins in a red algal nucleotide database (genome and transcriptome) using tBLASTn (*e*-value cutoff = 1e-5). The homologous protein sequences translated from the hit nucleotide sequences were collected using an in-house script. For each query sequence, the translated proteins corresponding to the three top bit-score hits and the three top-identity (query-hit identity) hits were incorporated into the single-gene ML tree building procedure described above. Distantly related homologs were manually identified and removed. Red algal sequences that were monophyletic with *G. sulphuraria* were considered to be orthologs.

## Results and Discussion

**Red algal tree of life**

We constructed single-gene alignments for a total of 298 one-to-one orthologous genes (98,494 amino acid positions in total) that are conserved in 15 red algal and 3 green algal taxa (see Methods). Analysis of the concatenated super-protein alignment under the CAT model led to a highly supported phylogenetic tree that received 1.00 posterior probability for all interior nodes (Fig. 1A). This tree confirmed the early split between Cyanidiophytina and Rhodophytina^4^^,^ 8 and monophyletic relationship between Bangiophyceae and Florideophyceae^4^^,^ 8. The relationships within Florideophyceae are consistent with previous analyses^10^^,^ 31 with Hildenbrandiophycidae (*Hildenbrandia*) in the basal position. Nemaliophycidae (*Palmaria*) is sister to the monophyletic group containing Corallinophycidae (*Calliarthron*) and Rhodymeniophycidae (*Chondrus*)^10^^,^ 31. The remaining non-seaweed mesophilic lineages formed a robust monophyletic group, with Stylonematophyceae in the basal position. Compsopogonophyceae formed a sister group to the monophyletic Porphyridiophyceae and Rhodellophyceae.

Concatenation-based analysis has previously been shown in some instances to result in inflated statistical support for incorrect topologies^32^ due to heterogeneity across genes and gene-specific evolution, such as gene duplication^33^. To minimize this problem, we used a tree summarization approach that does not rely on the concatenation of multiple single-gene alignments. This method takes a population of single-gene trees as input and estimates the species tree using a coalescence model^28^. This analysis led to the same tree topology (Fig. 1A) to the concatenation-based analysis with high bootstrap support for the monophyletic group comprising red seaweeds (bootstrap support = 100%) and non-seaweed mesophilic red algae (bootstrap support = 90%). The relationships among non-seaweed red algal lineages are however weakly supported (bootstrap support = 49-51%). Taken together, our phylogenomic analyses strongly support a separation between seaweeds and non-seaweed lineages at the base of mesophilic red algae (Fig. 1A).

**Red algal phylogenomics:**
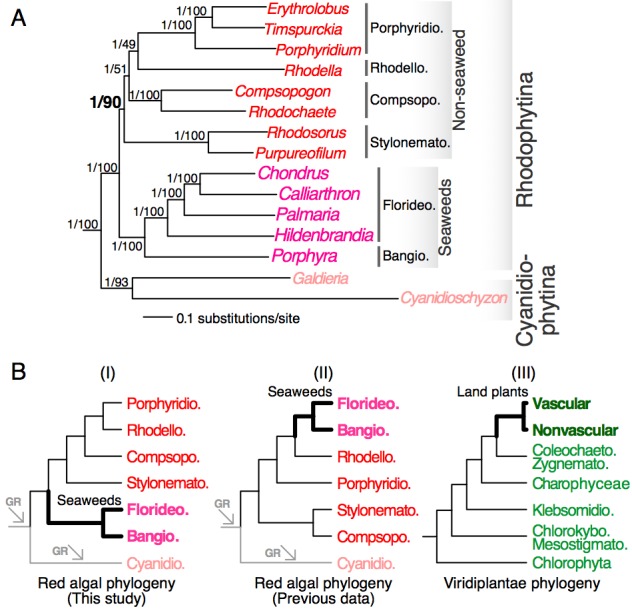
(A) A phylogenetic tree inferred from a concatenated 298-protein alignment. The outgroup species are not shown. Statistical supports (separated by a back slash) for each branch are derived from the super-protein analysis (posterior probability) and from the coalescence model-based analysis (bootstrap support). (B) Schematic representation of the positions of red seaweeds and land plants (thick branches) in red algae and Viridiplantae, respectively. The phylogenies are derived from this study (panel I), Scott et al (Ref. 6, panel II) and Leliaert et al. (Ref. 35, panel III). The arrows indicate genome reduction (GR). Bangiophyceae (Bangio.), Compsopogonophyceae (Compsopogo.), Cyanidiophyceae (Cyanidio.), Florideophyceae (Florideo.), Porphyridiophyceae (Porphyridio.), Stylonematophyceae (Stylonemato.), Coleochaetophyceae (Coleochaeto.), Chlorokybophyceae (Chlorokybo.), Klebsormidiophyceae (Klebsormidio.), Mesostigmatophyceae (Mesostigmato.), Zygnematophyceae (Zygnemato.).

**Parallel losses of MVA pathway**

To demonstrate the usefulness of this novel perspective on red algal phylogeny, we used the reference tree to elucidate the evolution of the isopentenyl pyrophosphate (IPP) biosynthetic pathway. IPP is the building block of isoprenoids that comprises a large diversity of lipids found in all three domains of life. In photosynthetic eukaryotes, two independent pathways exist to produce IPP, the cytosolic and peroxisome localized MVA pathway and the plastid MEP pathway^38^. Whereas the MEP pathway is conserved across many species, the MVA pathway has been lost in green algae (Chlorophyta)^38^ and in some red algal lineages such as *C.* *merolae* 16 and *P. purpureum* 12. Our analysis of red algal sequence data (see Methods) showed that the MEP pathway is present in all examined lineages. The minor gene losses that were found are most likely to be explained by missing data commonly associated with transcriptome datasets (Fig. 3, Appendix 2). In contrast, the MVA pathway is largely absent (3rd to 6th enzymes in the pathway, Fig. 2A) in most red algal lineages except the Stylonematophyceae (*Rhodosorus marinus* and *Purpureofilum apyrenoidigerum*) and *G. sulphuraria*. Presence of the MVA pathway in *G. sulphuraria* 39 and *Cyanidium caldarium* 40 is supported with genetic and biochemical evidence^39^^,^ 40. This result suggests that loss of MVA pathway is more widespread than previously thought. The red algal origin of the MVA genes in Stylonematophyceae is supported with phylogenetic data (see Methods). For example, in the phylogeny of HMG-CoA reductase (HMGR, Fig. 2B), *R. marinus* and *P. apyrenoidigerum* form a monophyletic group with and *Galdieria* species, whereas no other red algae were present in this clade. A similar pattern is found for other MVA pathway genes that were lost in most red algal species (Fig. 4, Appendix 3).

**MVA pathway in red algae:**
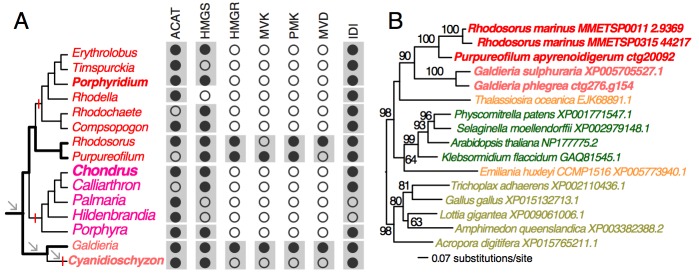
(A) The distribution of MVA pathway genes across red algal species. Black and open circles denote the presence and absence of the genes, respectively. For each gene, the gray boxes indicate gene presence for the corresponding classes. Arrows indicate genome reduction. Red vertical bars indicate gene losses. ACAT (acetyl-CoA acetyltransferase), HMGS (hydroxymethylglutaryl-CoA synthase), HMGR (3-hydroxy-3-methylglutaryl-CoA reductase), MVK (mevalonate kinase), PMK (phosphomevalonate kinase), MVD (mevalonate decarboxylase), IDI (isopentenyl-diphosphate delta-isomerase). (B) A ML tree of HMGR. The taxa in red color: red algae, green: Viridiplantae, orange: chromalveolates, brown: Opisthokonta.

Absence of the MVA pathway in all five sampled red seaweeds suggests it was most likely lost in their common ancestor. BLASTp searches (*e*-value cutoff = 10) against nucleotide databases (expressed sequence tag and transcriptome shotgun assembly) in NCBI did not return any significant hits to MVA pathway genes from Bangiophyceae and Florideophyceae. In addition, their losses in *C. merolae*, *P. purpureum*, and *C. crispus *that have both transcriptome and genome data available are well supported. Given the red algal phylogeny (Fig. 1A), these losses were unambiguously resulted from three parallel events (Fig. 2A). Under this scenario, the MVA pathway survived the ancient phases of genome reduction (arrows, Fig. 2A) and underwent gene loss more recently after the split of the seaweed and non-seaweed lineages. MVA pathway loss in *C. merolae *likely resulted from an additional phase of genome reduction specific to this lineage^30^ (Fig. 2A). The selective forces that led to the retention or loss of the MVA pathway across the mesophilic red algal lineages are presently unknown. Nonetheless, MVA pathway loss suggests that IPP biosynthesis is dependent on the plastid MEP pathway and requires transporters for the export of IPP from the plastid to the cytosol^38^. The MVA pathway was also lost in Chlorophyta (including most unicellular green algae)^38^ and *G. sulphuraria *is physiologically distinct from mesophilic species. For this reason, the discovery of possible MVA pathway-containing and -absent lineages among mesophilic red algae provides an algal model for studying the evolution of isoprenoid biosynthesis and intracellular trafficking among compartments.

## Conclusion

Our phylogenomic analyses resulted in a well-supported red algal phylogeny that provides new insights into the evolution of red seaweeds. Our results will allow more accurate reconstruction of evolutionary events (e.g., gene family evolution^2^ and molecular calibration^10^) and provide a framework to map the distribution of red algal functions and traits. Further efforts are needed to substantiate the relationships among non-seaweed mesophilic red algae with high quality genome data from these taxa^41^.

## Data Availability

The multi-protein alignment is available for download (ID: 20087) from TreeBASE (https://treebase.org).

## Competing Interests

The authors have declared that no competing interests exist.

## Corresponding Author

Huan Qiu, Department of Ecology, Evolution and Natural Resources, Rutgers University, New Brunswick, NJ 08901, USA.

E-mail: huan.qiu.bio@gmail.com

## Appendix 1

**Table 1:** Algal genome and transcriptome data used for the phylogenomic analysis

Classification	Species	Source	Data type	MMETSP ID
Seaweed	Hildenbrandia rubra	Ref. 2	Transcriptome	-
Seaweed	Palmaria palmata	Ref. 2	Transcriptome	-
Seaweed	Calliarthron tuberculosum	Ref. 14	Partial genome	-
Seaweed	Chondrus crispus	Ref. 13	Whole genome	-
Seaweed	Porphyra umbilicalis	Ref. 14	Transcriptome	-
Mesophiles	Purpureofilum apyrenoidigerum	Ref. 2	Transcriptome	-
Mesophiles	Rhodochaete pulchella	Ref. 2	Transcriptome	-
Mesophiles	Rhodosorus marinus	Ref. 11	Transcriptome	MMETSP0315
Mesophiles	Rhodella maculata	Ref. 11	Transcriptome	MMETSP0167
Mesophiles	Compsopogon coeruleus	Ref. 11	Transcriptome	MMETSP0312
Mesophiles	Erythrolobus australicus	Ref. 11	Transcriptome	MMETSP1353
Mesophiles	Timspurckia oligopyrenoides	Ref. 11	Transcriptome	MMETSP1172
Mesophiles	Porphyridium purpureum	Ref. 12	Transcriptome	-
Extremophiles	Galdieria sulphuraria	Ref. 15	Transcriptome	-
Extremophiles	Cyanidioschyzon merolae	Ref. 16	Whole genome	-
Green algae	Chlorella variabilis	Ref. 17	Whole genome	-
Green algae	Chlamydomonas reinhardtii	Ref. 18	Whole genome	-
Green algae	Micromonas pusilla	Ref. 19	Whole genome	-

## Appendix 2

**Distribution of the MEP pathway across red algal lineages:**
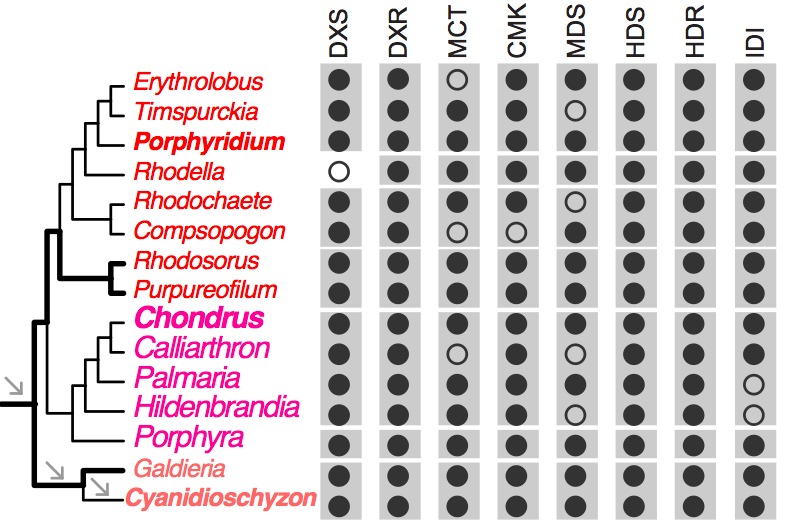
Black and open circles denote the presence and absence of the genes, respectively. For each gene, the gray boxes indicate the gene presence for the corresponding classes. DXS (1-deoxy-d-xylulose 5-phosphate synthase), DXR (1-deoxy-d-xylulose 5-phosphate reductoisomerase), MCT (2-C-methyl-d-erythritol 4-phosphate cytidylyltransferase), CMK (C-methyl-d-erythritol kinase), MDS (2-C-methyl-d-erythritol 2,4-cyclodiphosphate synthase), HDS (4-hydroxy-3-methylbut-2-en-1-yl diphosphate synthase), HDR (4-hydroxy-3-methylbut-2-en-1-yl diphosphate reductase), IDI (isopentenyl-diphosphate isomerase).

## Appendix 3

**ML trees for six MVA pathway genes:**
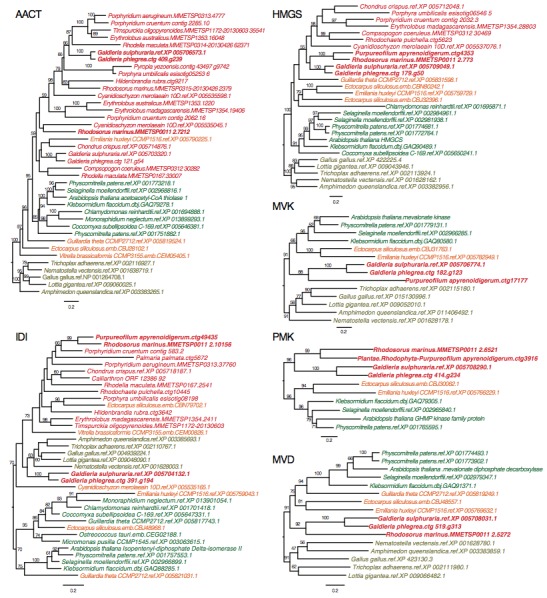
The taxa in red color: red algae, green: Viridiplantae, orange: chromalveolates, brown: Opisthokonta.
